# Surgical treatment strategies for gastroesophageal reflux after laparoscopic sleeve gastrectomy

**DOI:** 10.3389/fendo.2024.1463567

**Published:** 2024-10-29

**Authors:** Genzheng Liu, Pengpeng Wang, Shuman Ran, Xiaobin Xue, Hua Meng

**Affiliations:** China-Japan Friendship Hospital (Institute of Clinical Medical Sciences), Chinese Academy of Medical Sciences & Peking Union Medical College, Beijing, China; Department of General Surgery and Obesity and Metabolic Disease Center, China–Japan Friendship Hospital, Beijing, China

**Keywords:** laparoscopic sleeve gastrectomy, gastroesophageal reflux, surgical treatment, refractory gastroesophageal reflux, revision surgery

## Abstract

Bariatric surgery has emerged as an effective therapeutic approach for combating obesity. As the most commonly performed bariatric surgery, laparoscopic sleeve gastrectomy (LSG) has a long-term and effective outcome in weight reduction. However, studies have reported an increased incidence of gastroesophageal reflux disease (GERD) among patients after LSG. For those who fail to respond to conventional oral acid-suppressing medication, surgical intervention comes into consideration. The most commonly performed revisional surgery for sleeve gastrectomy is the Roux-en-Y gastric bypass, which can effectively alleviate the symptoms of reflux in patients and also continues to promote weight loss in patients who have not achieved satisfactory results or have experienced weight regain. In addition to this established procedure, innovative techniques such as laparoscopic magnetic sphincter augmentation (MSA) are being explored. MSA is less invasive, has good reflux treatment outcomes, and its safety and efficacy are supported by the literature, making it a promising tool for the future treatment of gastroesophageal reflux. This article also explores the role of endoscopic interventions for GERD treatment of post-sleeve gastrectomy patients. Although these methods have shown some therapeutic effect, their efficacy still requires further study due to a lack of support from more clinical data. For patients with preoperative hiatal hernia or gastroesophageal reflux symptoms, some experts now consider performing LSG combined with hiatal hernia repair or fundoplication to alleviate or prevent postoperative reflux symptoms. Both of these surgical approaches have demonstrated favorable outcomes; however, the addition of fundoplication requires further investigation regarding its long-term effects and potential postoperative complications. This article gathers and examines the current laparoscopic and endoscopic treatments for refractory gastroesophageal reflux following LSG, as well as the concurrent treatment of LSG in patients with preoperative gastroesophageal reflux or hiatal hernia.

## 1 Introduction

Obesity has emerged as a critical global health concern. Between 1975 and 2014, the prevalence of obesity among adult men surged from 3.2% to 10.8%, while the prevalence among women increased from 6.4% to 14.9% (1). In China, the percentage of overweight adults has climbed to 34.8%, with an obesity rate of 14.1% (2). Bariatric surgery, with its significant weight loss outcomes, long-term stability, and high remission rate for obesity-related complications, has increasingly become one of the weight loss options for obese individuals. Laparoscopic sleeve gastrectomy (LSG) has become the most commonly performed bariatric procedure due to its relative simplicity and positive outcomes. In the United States, LSG accounts for approximately 57.4% of all bariatric surgeries (3). Despite its popularity, recent research suggests that LSG may exacerbate postoperative gastroesophageal reflux or lead to *de novo* reflux episodes (4). A meta-analysis encompassing 22 studies revealed a 35% incidence of gastroesophageal reflux following LSG (5). In the long-term follow-up after LSG, the incidence of new-onset gastroesophageal reflux is 20.0% to 24.8% (6, 7). However, some studies have reported even higher rates, with the incidence of postoperative reflux potentially ranging from 50% to 53.8%, and the rate of new-onset reflux reaching up to 42.3% to 73% (8–10). This discrepancy may be attributed to demographic differences or variations in dietary habits.

Currently, when addressing gastroesophageal reflux in patients post-LSG, the predominant approach, considering safety and the desire to minimize postoperative patient trauma, remains focused on dietary and lifestyle adjustments, in conjunction with the administration of antacid and acid-suppressing pharmaceuticals. However, there is no unified treatment guideline in the academic community for dealing with persistent gastroesophageal reflux that is unresponsive to long-term acid suppression therapy after LSG.

This article reviews the possible mechanism and the currently available treatment strategies for gastroesophageal reflux after LSG, including non-surgical treatment, surgical treatment and concurrent surgical treatment.

## 2 Mechanisms of gastroesophageal reflux after LSG

The potential factors contributing to gastroesophageal reflux after LSG surgery include reduced gastric compliance, elevated intra-gastric pressure, and the disruption of the anti-reflux barrier, such as an enlarged His angle and decreased lower esophageal sphincter pressure. Other factors may include the presence of a hiatal hernia, gastric sleeve torsion, stenosis, etc. (11) Quero et al. (12) employed magnetic resonance imaging, high-resolution manometry, and dynamic pH impedance measurement to evaluate the structure and function of the gastroesophageal junction and stomach before and after LSG. Their research revealed that the His angle increased from 36° to 51° following LSG, with 78% of patients exhibiting an enlarged His angle. Postoperatively, the average length of the lower esophageal sphincter decreased by 1cm, and the mean intra-gastric pressure rose from 21.3 mmHg preoperatively to 33.5 mmHg postoperatively. Similar results have been reported by Mion, Balla, Poggi, and others (13–15). Furthermore, studies have indicated that the morphology of the residual stomach after LSG also plays a role in reflux dynamics (16).

## 3 Non-surgical treatment of GERD after LSG

For patients with gastroesophageal reflux who have not undergone surgery, conventional treatment methods include: 1. Dietary and lifestyle interventions, which involve avoiding foods that may trigger reflux (such as coffee, alcohol, chocolate, high-fat foods, etc.) and highly irritating foods (such as citrus, carbonated beverages, spicy foods, etc.), as well as losing weight, quitting smoking, elevating the head of the bed, and avoiding lying flat after meals. 2. The use of antacid and gastric mucosal protective agents, such as aluminum magnesium carbonate suspension. 3. The use of acid suppressants, including H2 receptor antagonists (such as cimetidine), proton pump inhibitors (such as omeprazole), and potassium-competitive acid blockers (such as vonoprazan) (17).

In the study by Peterli et al. (18), at the five-year follow-up, out of 101 patients who underwent LSG, 7 experienced reflux esophagitis that was unresponsive to proton pump inhibitor (PPI) treatment, and 1 had developed *de novo* Barrett mucosa. All of these patients eventually converted to RYGB. In another study by Paulina et al. (19), a ten-year follow-up found that as many as 64.4% of patients still required oral PPIs after LSG. For patients who remain dependent on medication long-term or for those whose symptoms are not adequately controlled by drugs, it becomes imperative to explore additional surgical or endoscopic intervention strategies.

## 4 Surgical treatment of GERD after LSG

### 4.1 Roux-en-Y gastric bypass

The most common reason for revision surgery after LSG is poor weight loss, weight regain, or severe gastroesophageal reflux, with RYGB being the most frequently performed revisional procedure following LSG (20). Both the American Gastroenterological Association and the American Society for Gastrointestinal and Endoscopic Surgeons consider RYGB to be the preferred surgery for obese patients with gastroesophageal reflux (21, 22). In a study by Mandalosso et al. (23), 53 patients were monitored for an average of 39 months to assess the changes in esophageal and extraesophageal symptoms before and after RYGB. The results showed that 83% of patients with typical reflux symptoms prior to surgery experienced significant improvement postoperatively.

Huynh et al. (24) utilized the Gastroesophageal Reflux Disease - Health-Related Quality of Life (GERD-HRQL) score to evaluate the quality of life in 41 patients who underwent RYGB following LSG. The average GERD-HRQL score plummeted from 31.5 before the revision surgery to 5.6 at 6 months post-RYGB, and it still remained 7.3 at 15 months. In a retrospective study by Felsenreich and others (25), among the 45 patients who converted from LSG to RYGB, 36 had preoperative GERD, and 6 had Barrett’s esophagus. Following the RYGB, Barrett’s esophagus was fully resolved in 4 patients, and symptoms of gastroesophageal reflux improved in 23 patients (63.9%). A similar study by Dayan (26) found that out of 47 patients with GERD who underwent RYGB as a revisional procedure after LSG, 43 (91.5%) experienced relief from their reflux symptoms. Insufficient weight loss is also a critical indication for revisional surgery following RYGB. In the study by Antonio et al. (27), the percentage of excess weight loss at 1, 3, and 5 years post-bypass was 40.3%, 34.3%, and 23.2%, respectively, for the group with poor weight reduction. In a separate study enrolling 97 individuals, those who underwent RYGB revisional surgery achieved an average weight loss of 11.1 ± 12.9 kg. For patients who underwent surgery to address reflux, 80.2% experienced overall symptom improvement following the revision, and 19.4% were able to cease PPI therapy postoperatively. However, the majority of patients (80.5%) still required oral PPIs at the last follow-up (average 16.5 ± 19.56 months). The incidence of complications classified as Clavien–Dindo grade III or higher was 7.21%, including grade IV complications accounting for 2.06% of the cases (with an average follow-up of 16.5 ± 19.56 months) (27, 28). In contrast, the reported short-term complication rate for primary RYGB surgery is 6.3% (29), and the 10-year complication rate is 24.4%, with an incidence of Clavien–Dindo grade III or higher complications at 18.5% (19). However, the sample sizes for RYGB revisional surgery studies are relatively small, and there is a scarcity of longer-term follow-up data. Consequently, further research is needed to compare the incidence of complications.

It is evident that RYGB has a favorable therapeutic effect in the treatment of gastroesophageal reflux and insufficient weight loss after LSG. Moreover, the complication rate of RYGB revisional surgery does not appear to be significantly higher than that of the primary RYGB. For patients who continue to struggle with refractory reflux symptoms after LSG, RYGB remains the preferred option for surgical revision. However, it is worth noting that some studies have identified an increased likelihood of experiencing weak acid reflux following RYGB (30).

### 4.2 Magnetic sphincter augmentation

MSA is a novel anti-reflux surgical technique, a method that utilizes the LINX Reflux Management System to achieve the goal of preventing reflux. The LINX device is a ring-like construct formed from a series of titanium beads, each embedded with a magnetic core and interconnected by independent titanium arms. This device is implanted laparoscopically around the lower esophageal sphincter to enhance its capability (31).

A meta-analysis encompassing three studies has revealed that patients who underwent MSA experienced an average reduction of 17.5 points in their GERD-HRQL scores, signifying MSA’s viability as a treatment for refractory GERD following bariatric surgery (32). In a clinical study by Patel et al. (33) that included 22 individuals, 82% of patients were able to cease using acid-suppressing medication after MSA, with average postoperative GERD-HRQL scores dropping from 43.8 preoperatively to 16.7 postoperatively, 77% of patients were “satisfied” with the MSA surgery, while 14% were “dissatisfied.” The main reason for dissatisfaction was the persistent need for acid suppressants to control reflux symptoms, and a few other patients experienced dysphagia after MSA. Other studies have also reported similar results, showing significant improvements in GERD-HRQL scores after MSA, with proton pump inhibitor discontinuation rates ranging from 69.2% to 90.0% (34–36). The study by Khaitan et al. (37) noted that common adverse events following MSA included dysphagia (16.7%), pain (10.0%), and nausea (6.7%). The research also compared the treatment differences of MSA surgery between patients with a history of weight loss surgery and those without gastric surgery, suggesting similar therapeutic effects in both groups (35, 36).

Based on the current results, MSA is a safe and effective surgical treatment for refractory gastroesophageal reflux following weight loss surgery. It can effectively alleviate reflux symptoms, allowing the majority of patients to avoid long-term oral acid suppression medication and improve their quality of life.

### 4.3 Antireflux mucosectomy

Antireflux mucosectomy is an endoscopic treatment technique first reported by Inoue and colleagues for the treatment of refractory GERD. It involves the use of endoscopic mucosal resection or endoscopic submucosal dissection to remove mucosal tissue at the gastroesophageal junction. As the mucosa heals, submucosal fibrosis and scarring develop, creating a postoperative antireflux barrier. Their study also suggests that ARMs may effectively improve symptoms and DeMeester scores in patients with GERD (38).

Zhu and colleagues (39) have reviewed the therapeutic outcomes of ARMs in recent studies. Analysis of six studies that recorded GERD-HRQL scores revealed varying degrees of improvement in patients following ARMs. In seven studies involving 24-hour esophageal pH monitoring, DeMeester scores significantly improved postoperatively, and the average time of esophageal acid exposure was greatly reduced. These findings confirm the efficacy of ARMs in the treatment of refractory GERD. They also highlighted common postoperative complications, mainly including dysphagia (11.4%) and bleeding (5%).

In a case study, Patil et al. (40) employed ARMs to treat a patient experiencing refractory GERD after LSG. The patient’s DeMeester score dramatically decreased from 159 to 13.8, and the 24-hour pH measurement of acid reflux time was reduced from 25% to 4.5%. Additionally, the GREDQ score fell from 10 to 7, allowing for the discontinuation of acid suppressants postoperatively. Debourdeau and colleagues (41) found similar results in their study of six patients who underwent ARMs after bariatric surgery, with the average GERD-HRQL score dropping from 30.6 to 6.8 at three months postoperatively. However, three patients still required ongoing acid suppressant therapy, and complications such as esophageal stricture and gastrointestinal bleeding were observed in one patient each.

ARMs demonstrate promising efficacy in the management of refractory GERD. While more clinical evidence is needed to support its use in patients with refractory GERD following weight loss surgery, the current studies suggest that ARMs can be an effective treatment option. Nonetheless, caution must be exercised to monitor and manage potential postoperative complications.

### 4.4 Endoscopic radiofrequency therapy

Endoscopic radiofrequency therapy is a treatment method that utilizes the Stretta device, which primarily consists of a catheter with four nickel-titanium needle electrodes and a guide wire. During treatment, the device is positioned at the gastroesophageal junction, and heat energy is released within a 2cm range above and below the squamocolumnar junction, with the temperature maintained at 85°C. To prevent overheating, cold water is used to cool the tissue, ensuring that the mucosal temperature stays below 30°C for a period of 2 minutes. This process ultimately induces scar formation in the lower esophageal sphincter through heat stimulation, thereby increasing the pressure in the lower esophageal sphincter. Additionally, the radiofrequency energy can disrupt the intramuscular vagal ganglia in the esophagus, preventing vagally induced transient lower esophageal sphincter relaxation, thus achieving the therapeutic goal (42, 43).

In a study that followed 83 patients for 4 years, the proportion of patients using acid suppressants decreased from 100% at baseline to 29.4% at 12 months, 12.1% at 36 months, and 13.75% at 48 months after radiofrequency treatment. Concomitantly, there was a marked improvement in both the symptoms score and the quality of life score for gastroesophageal reflux (44).

In a retrospective study involving 15 patients, the efficacy of radiofrequency treatment for gastroesophageal reflux after LSG was assessed. At the six-month post-treatment mark, a majority of patients (66.7%) expressed dissatisfaction, and only a fifth (20%) had ceased using acid suppressants. Additionally, two patients (13.3%) required a subsequent RYGB surgery at eight months post-treatment to address persistent reflux symptoms (45). Therefore, the therapeutic role of radiofrequency treatment in patients with reflux after LSG requires further investigation.

### 4.5 Other treatment modalities

A study comparing the conversion from LSG to One-Anastomosis Gastric Bypass (OAGB) versus RYGB surgery revealed that OAGB may offer superior outcomes in terms of acid exposure and DeMeester scores, even though it was associated with a higher prevalence of reflux symptoms in the OAGB group (25). Additional studies suggest that OAGB might be as effective as RYGB in addressing reflux following LSG. Data from Rheinwalt’s research indicate that the rates of GERD remission after converting from LSG to RYGB and OAGB were 89% and 87%, respectively (46). Dayan et al. (26) found that 77.4% of patients who underwent conversion to OAGB after LSG experienced a resolution of reflux symptoms and were able to discontinue acid suppressants, with their average GERD-HRQL score plummeting from 9.6 preoperatively to 1.7 postoperatively.

Endoscopic interventions that are commonly used for GERD in patients who have not had gastric surgery have not yet been widely adopted for the treatment of post-LSG reflux. These include procedures such as transoral incisionless fundoplication and transoral endoscopic cardial plication. Given the uncertain safety profile of these methods, further study is required to determine their safety and therapeutic effectiveness in the context of post-LSG reflux management.

## 5 Concurrent surgical treatment and prevention of reflux during LSG

### 5.1 LSG combined with hiatal hernia repair

Excessive body weight is significantly associated with the presence of hiatal hernia and esophagitis (47). Furthermore, central obesity and hiatal hernia can lead to an increase in GERD (48). A meta-analysis of 18 studies suggests that LSG combined with Hiatal Hernia Repair (HHR) results in a 68% reduction in GERD symptoms, as well as significant improvements in esophagitis and GERD- HRQL. However, the article also notes that there is no significant difference between LSG + HHR and LSG alone in terms of new-onset GERD, with postoperative incidence rates of new GERD at 12.0% and a recurrence rate of hiatal hernia at 11.0% (49). Additionally, another study that included 91 patients and completed a 7-year follow-up found that among patients with preoperative gastroesophageal reflux, 60% experienced relief from their symptoms. Nevertheless, 30.6% of patients reported postoperative reflux symptoms, with 15.9% experiencing persistent GERD and 14.8% experiencing new-onset GERD (50).

In the study by Perez et al. (51), they utilized Propensity Score Matched Analysis to examine patients from The Metabolic and Bariatric Surgery Accreditation and Quality Improvement (MBSAQIP) database. They found that LSG + HHR had a similar risk of death, postoperative bleeding, leakage, or reoperation within 30 days after surgery when compared to LSG alone. However, the risk of postoperative pneumonia (0.45% vs 0.15%) and readmission rates (4.69% vs 3.58%) were higher after LSG+HHR.

HHR encompasses three primary techniques: posterior repair with mesh (PRM), posterior repair (PR), and anterior repair (AR). Ehlers and colleagues (52) conducted an analysis of data from the Michigan Bariatric Surgery Collaborative (MBSC) spanning from 2008 to 2019, revealing that PR was the most frequently performed procedure, constituting 78% of the cases. The severity of heartburn at baseline was assessed using the GERD- HRQL scale, with the PRM group exhibiting the highest scores (PRM 1.40 versus PR 1.20 versus AR 0.99). However, the PR cohort had the lowest average heartburn severity score at one year postoperatively (PR 0.81 vs PRM 0.84 vs AR 0.96). Patients across all three surgical groups reported high levels of satisfaction at the one-year postoperative follow-up, with no statistically significant differences in satisfaction among the groups. Additionally, there were no significant differences in the incidence of bleeding, leakage, or surgical complications at 30 days postoperatively across the three cohorts. In a separate study based on the same database covering the period from 2015 to 2019, Hider and associates (53) focused solely on the comparison between PR and AR. They found that patients undergoing PR had a higher rate of improvement in GERD symptoms (69.5% vs 64.0%) and a lower rate of new symptoms at one year (28.2% vs 30.2%). Conversely, patients receiving AR had higher rates of bleeding and readmission.

In conclusion, the combination of LSG and HHR has been shown to substantially reduce GERD symptoms in patients with a minimal risk of complications. Performing HHR concurrently with LSG is deemed a safe surgical practice, and within the HHR techniques, posterior repair seems to be the preferred approach. A panel of 50 experts from across 25 nations engaged in a discourse on LSG and GERD, with 80% of the experts supporting the concurrent repair of large hiatal hernias during LSG, and 66.7% supporting the concurrent repair of small hiatal hernias (54).

### 5.2 LSG combined with fundoplication

Fundoplication has long been the standard approach for surgical treatment of GERD. This procedure involves using either a portion of the stomach fundus (Dor 180°, Toupet 270°) or the entire fundus (Rossetti, Nissen 360°) to wrap around the lower esophageal sphincter (LES), providing structural support to enhance its ability to prevent reflux. Studies conducted by Capua and associates have shown that the combination of LSG and Rossetti fundoplication can significantly elevate LES pressure in patients following surgery (55). In a survey of 50 experts, 77.3% reported using LSG along with an anti-reflux procedure (either anterior or posterior fundoplication) in patients with GERD symptoms (54).

Olmi and colleagues performed surgery using LSG combined with a modified Rossetti fundoplication on 220 patients, of which 68.5% had preoperative reflux symptoms. Following the procedure, 98.5% of the patients reported no reflux symptoms and were not reliant on PPI medication. Among those with preoperative esophagitis, 96.9% experienced relief, and all four patients with Barrett’s esophagus showed improvement. The incidence of Clavien–Dindo grade III or higher complications was 6.9%, primarily due to fundus perforations (56). In another study involving 56 patients, the postoperative GERD outcomes of LSG and LSGFD were compared. Patients without preoperative reflux symptoms were placed in the LSG group, while those with reflux symptoms were assigned to the LSGFD group. At 12 months postoperatively, the incidence of new-onset GERD following LSG was 52.2%, which reduced to 30.4% at an average follow-up of 34 months. In the LSGFD group, 86.4% experienced relief from reflux symptoms at 12 months, and 90.9% did so at an average follow-up of 34 months. There were no notable disparities between the two groups in terms of weight loss and postoperative complications (57).

In a meta-analysis encompassing five studies, researchers reviewed the current evidence and outcomes of LSGFD. They discovered that LSGFD resulted in superior GERD relief postoperatively but may lead to a lower percentage of total weight loss and a higher incidence of postoperative complications (OR=2.56) (58).

LSGFD is evidently effective in the prevention and treatment of GERD, but it also comes with an increased risk of postoperative complications. Currently, there is a scarcity of literature comparing the outcomes of LSG and LSGFD, and the clinical application of LSGFD requires careful assessment of the risks versus benefits by the surgeon. In the comparison between LSG+HHR and LSGFD, both procedures are effective in alleviating and preventing GERD following LSG. LSGFD demonstrates a stronger advantage in managing reflux, but it also has a higher overall complication rate (59).

### 5.3 The impact of LSG surgical technique

The LSG surgical procedure can also impact postoperative GERD. Currently, the main disagreement among surgeons focuses on the distance between the resection line and the pylorus, as well as the sizes of the bougie.

A meta-analysis based on randomized controlled trials compared four RCT studies that described post-operative GERD and the distance between the resection line and the pylorus. It found that, in the late postoperative period, GERD was significantly reduced in the group where the resection was performed 6cm from the pylorus, compared to 2cm from the pylorus (OR=0.40) (60). However, an earlier meta-analysis showed no statistical difference in the incidence of new-onset GERD between antral resection (with the staple line starting 2-3 cm from the pylorus) and antral preservation (>5 cm from the pylorus) (61). Another study, based on an observational cohort, a shorter distance to the pylorus was found to be a predictor of postoperative GERD relief, while a shorter distance to the angle of His was a risk factor for new-onset GERD. But they did not find a correlation between the distance to the pylorus and the occurrence of GERD (62). Currently, there is still controversy over how far from the pylorus the resection should begin. Forty-one experts from China, Japan, and South Korea discussed postoperative GERD following LSG in Shanghai (63), with 70.7% of the experts agreeing that starting the resection 4-6 cm from the pylorus and reasonably preserving the antrum during surgery could effectively reduce the incidence of postoperative GERD.

Additionally, the choice of bougie size is quite controversial. In a meta-analysis conducted by Yao Wang and associates (64), bougie sizes were classified into two groups: less than or equal to 36Fr, and greater than 36Fr. They found that the smaller bougie group had better weight loss outcomes without increasing the risk of postoperative leaks or GERD. In another network meta-analysis, the bougie sizes were divided into four groups: XL (> 40 Fr), L (36–40 Fr), M (33–36 Fr), and S (< 32 Fr) (65). They discovered that the S and M size bougies were more effective in reducing excess weight, and the M size bougie had lower rates of postoperative leaks and overall complications. However, an earlier systematic review that included 112 studies found that bougies ≥40 Fr reduced the risk of leaks (OR = 0.53). The distance from the pylorus was found to have no impact on leaks or the percentage of excess weight loss (66). In the Shanghai conference (63), considering that excessive resection of the fundus may lead to rapid gastric emptying, affecting patients’ dietary control and weight loss outcomes, 85.4% of experts agreed to recommend the use of 36~38Fr bougies to ensure surgical effectiveness while reducing the incidence of postoperative GERD.

## 6 Conclusion

The high incidence of gastroesophageal reflux after LSG has become a significant challenge for post-operative patients. Currently, the primary approach to managing reflux after LSG is through conservative medical interventions. When faced with persistent gastroesophageal reflux that does not respond to acid suppressants for over three months, the prevailing recommendation within the medical community is to transition to Roux-en-Y gastric bypass (RYGB) surgery. The revised RYGB surgery has proven effective in controlling post-LSG reflux symptoms and enhancing patients’ quality of life. In recent times, innovative surgical techniques have been gaining traction as a means to address the symptoms of refractory gastroesophageal reflux in patients after LSG. Laparoscopic magnetic sphincter augmentation, for instance, has shown promise in mitigating acid reflux symptoms and reducing the duration of esophageal acid exposure. Additionally, other endoscopic treatments have yielded positive therapeutic outcomes. Nevertheless, the long-term safety and effectiveness of these interventions for post-LSG reflux remain to be fully validated through future research. For patients who are found to have hiatal hernia during preoperative evaluation, concurrent repair of the hiatal hernia during LSG can be contemplated. This approach significantly reduces the probability of postoperative GERD and has a relatively low incidence of postoperative complications, making it a safe and effective surgical option. For those with preoperative gastroesophageal reflux, LSGFG can be considered to forestall the onset of postoperative GERD. However, this procedure may introduce additional surgical risk, and the decision to proceed with LSGFG should be made following thorough consideration by the surgical team.
