# Corrigendum: Surgical treatment strategies for gastroesophageal reflux after laparoscopic sleeve gastrectomy

**DOI:** 10.3389/fendo.2025.1630859

**Published:** 2025-06-05

**Authors:** Genzheng Liu, Pengpeng Wang, Shuman Ran, Xiaobin Xue, Hua Meng

**Affiliations:** ^1^ China-Japan Friendship Hospital (Institute of Clinical Medical Sciences), Chinese Academy of Medical Sciences & Peking Union Medical College, Beijing, China; ^2^ Department of General Surgery and Obesity and Metabolic Disease Center, China–Japan Friendship Hospital, Beijing, China

**Keywords:** laparoscopic sleeve gastrectomy, gastroesophageal reflux, surgical treatment, refractory gastroesophageal reflux, revision surgery

In the published article, there was an error in the affiliation for Genzheng Liu. As well as having affiliation(s) “Department of General Surgery and Obesity and Metabolic Disease Center, China–Japan Friendship Hospital, Beijing, China”, this author should also have “China-Japan Friendship Hospital (Institute of Clinical Medical Sciences), Chinese Academy of Medical Sciences & Peking Union Medical College, Beijing, China”.

The authors apologize for this error and state that this does not change the scientific conclusions of the article in any way. The original article has been updated.

